# Enjoyment as a Predictor of Exercise Habit, Intention to Continue Exercising, and Exercise Frequency: The Intensity Traits Discrepancy Moderation Role

**DOI:** 10.3389/fpsyg.2022.780059

**Published:** 2022-02-18

**Authors:** Diogo S. Teixeira, Filipe Rodrigues, Luis Cid, Diogo Monteiro

**Affiliations:** ^1^Faculty of Physical Education and Sport (ULHT), Lusófona University of Humanities and Technologies, Lisbon, Portugal; ^2^Research Center in Sport, Physical Education, and Exercise and Health (CIDEFES), Lisbon, Portugal; ^3^ESECS, Polytechnic of Leiria, Leiria, Portugal; ^4^Quality of Life Research Center (CIEQV), Santarém, Portugal; ^5^Research Center in Sport, Health and Human Development (CIDESD), Vila Real, Portugal; ^6^Sport Science School of Rio Maior (ESDRM), Polytechnic Institute of Santarém, Santarém, Portugal

**Keywords:** exercise, intensity, enjoyment, intention, habit, moderation

## Abstract

Given the need to explore the factors that can account for a better understanding of the intention-behavior gap in exercise practice in health club settings, and considering the emergence of hedonic assumptions related to exercise adherence, this cross-sectional study aimed to test the moderation effect of the intensity traits agreement/disagreement in three relevant outcomes of exercise enjoyment: exercise habit, intention to continue exercising, and exercise frequency. A sample consisted of 273 exercisers (male = 127; *M*_age_ = 36.21; *SD* = 11.29) enrolled in nine health clubs who voluntarily fulfilled a battery of questionnaires. All analyses were performed using SPSS v. 23.0/PROCESS v. 3.5. The results of the study presented a moderation effect of exercise intensity traits agreement on three relevant enjoyment outcomes: exercise habit, intention to continue exercising, and exercise frequency. No relevant results emerged from intensity traits disagreement. The results suggest that assessing and tailoring exercise prescription and supervision in order to customize exercise intensity may influence future exercise participation.

## Introduction

Decades of reports have shown that health clubs register high dropout rates, particularly in the first 3 to 6 months of practice (Edmunds et al., 2007; Buckworth et al., 2013; Sperandei et al., 2016). Given that globally, these are one of the most relevant contexts of exercise practice (International Health Racquet and Sportsclub Association, 2020; EC, 2018) promoting sustainable and long-term exercise adherence is paramount.

As a means to understand exercise adherence and dropout, several theoretical approaches have been used to measure and test motivational and cognitive determinants of individuals’ behavior. One aspect that has been consistently reported as relevant for exercise participation is enjoyment (Rhodes and Kates, 2015; Chen et al., 2020; Klos et al., 2020), understood as a subjective experience that depicts generalized feelings of pleasure and satisfaction (Moore et al., 2009; Nielsen et al., 2014). Enjoyment can, in turn, substantially affect individual perceptions of the activity, thus reinforcing it (when perceived as interesting or pleasant) or avoiding it (when perceived as unpleasant, uninteresting, or boring), influencing exercise commitment and engagement (Jekauc and Brand, 2017; Teixeira et al., 2021a).

A few recent approaches have brought new insights and considerations that highlight pleasure and enjoyment as relevant factors that can explain and support exercise behavior. For example, the Affect and Health Behavior Framework (AHBF; Williams and Evans, 2014; Stevens et al., 2020) divides affective correlates and determinants (e.g., enjoyment) into four categories which, eventually, can help explain variables related to health behavior. Another approach is the Affective-Reflective Theory (ART; Brand and Ekkekakis, 2018) that postulates that physical inactivity and exercise can be explained through dual-process theory perspectives that underline automatic associations to pleasurable states. One more recent paradigm, also grounded in dual-process theories, is called the Physical Activity Adoption and Maintenance model (PAAM; Strobach et al., 2020). It presents and identifies predictors of physical activity structured by explicit (i.e., reflective, deliberate) and implicit (i.e., affective, automatic) processes. Particularly, in the PAAM model, these processes are postulated to have direct, moderated, and interaction effects on physical activity. All in all, theoretical approaches grounded in hedonic assumptions have considerably reinforced the need for further development of research in various physical activity settings.

In health clubs, enjoyment seems to be a relevant predictor of the intention to continue exercising, exercise habit, and adherence, which are relevant outcomes capable of promoting behavior sustainability (Raedeke, 2007; Calder et al., 2020; Rodrigues et al., 2020). Intention, for example, has been proposed to be a proximal determinant of behavior enactment (Ajzen, 1991; Armitage, 2005). Although it has been proposed to not fully explain individual behavior as a separate variable, in literature it still does emerge as a relevant construct related to exercise practice (Norman et al., 2000; Jekauc et al., 2015; Gomes et al., 2018). It has been suggested that higher levels of enjoyment can manifest some effect on intentions, which could be related to exercise commitment and persistence (Rodrigues et al., 2020), being a relevant factor for understanding variables related to the intention-behavior gap. The AHBF and the PAAM model, for example, present behavioral intention as a result of reflective processes, which can receive influences (generally indirect) from affective processing (e.g., enjoyment) (Stevens et al., 2020; Strobach et al., 2020).

As for habit, it has been defined as a learning sequence of acts that can result in automatic responses linked to specific cues, and has been associated with exercise behavior in health clubs (Kaushal and Rhodes, 2015; Weyland et al., 2020; Feil et al., 2021). In the PAAM model, for instance, it is suggested that the repetition of a behavior in the same context can shift the behavior gradually from explicit to implicit control processes (thus aiding habit formation), and that this shift can be supported by positive affective reactions to physical activity. Therefore, affect and enjoyment can help habit formation and may be particularly relevant for exercise maintenance, thus reinforcing the need to understand possible factors and mechanisms that may account for the enjoyment effect.

Another relevant aspect that has been proven to influence pleasurable experiences is exercise intensity. As reported in several studies, increases in exercise intensity are usually related to more pleasurable experiences, up until the point where intensity tends to present reduced pleasure and increase displeasure (Ekkekakis et al., 2011; Evmenenko and Teixeira, 2020). Moreover, the point where exercise intensity tends to negatively influence the pleasure/displeasure ratio appears to have some inter-individual variability (Ekkekakis et al., 2011; Ladwig et al., 2017), highlighting the urge to better understand how this turning point can influence pleasurable experiences, enjoyment development, as well as promote adherence.

On the same topic related to the promotion of pleasurable experiences during exercise, some studies have tested the role of preference (i.e., predisposition to select a particular intensity level) and tolerance (i.e., individual ability to continue exercising at a defined level of intensity) as intensity traits relevant to the individual understanding of how intensity is related to exercise pleasurable responses (Ekkekakis et al., 2005; Box and Petruzzello, 2020; Teixeira et al., 2021a). In health club settings, some preliminary evidence suggests that the intensity traits may have a relevant role in the comprehension of several cognitive, behavioral and emotional outcomes. Particularly, Teixeira et al. (2021b) and Faria et al. (2021) have found positive associations between both traits and exercise frequency, habit, subjective vitality and well-being; Box and Petruzzello (2020) and Teixeira et al. (2021a) have demonstrated positive associations between the intensity traits and enjoyment. Moreover, it has been suggested that the intensity traits can modulate individual affective responses to an exercise regimen or a particular activity (Box and Petruzzello, 2020; Andrade et al., 2021) which, all in all, reveals the impact of intensity adjustments on exercise adherence.

Considering the diversity of exercise modes and dynamics in health clubs, it seems plausible to hypothesize that individual preference and tolerance might not always be contemplated in exercise prescription or supervision (Teixeira et al., 2021b). On one hand, gym dropout issues in the first months of practice have been suggested to be related to poor professional follow-up and supervision, which may also account for inadequate management of pleasure/displeasure in the first weeks or months of exercise (Rand et al., 2020; Faria et al., 2021). On the other hand, even in regular exercisers, interpersonal dynamics have been reported as a relevant factor for exercise adherence, and the steps taken by the professionals to make individual adjustments to the training characteristics are proposed to modulate the exercise behavior (Rodrigues et al., 2018, 2019). Hence, all exercisers can be at some point more or less prone to have their pleasure/displeasure ratio affected. In a setting where exercise professionals are able to monitor and adjust training variables, the understanding of the potential role of the intensity traits in exercise practice may be relevant for the intended promotion of exercise adherence.

Several authors have highlighted the importance of targeting enjoyment development as a proxy for exercise adherence and maintenance (Rhodes et al., 2019; Calder et al., 2020; Chen et al., 2020). However, less attention has been given to the very factors and mechanisms that may influence enjoyment effect on related outcomes. For instance, a professional may suggest a group class that suits the exerciser’s needs and that is aligned with his perception of a pleasant and enjoyable activity, but that is not congruent with the intensity preference/tolerance (e.g., an advanced high intensity training class); or, in personal training, the progression to a new mesocycle that presents significant changes to previously enjoyable exercise intensity, may alter this perception and cause different outcomes. Therefore, an enjoyable activity may present distinct effects on adherence related factors due to the level of the intensity traits agreement/disagreement perceived by the exerciser. This may modulate an individual’s future practice through an avoidance-approach effect grounded in hedonic assumptions, which may reinforce or reduce exercise behavior and intentions (Watson et al., 1999; Stevens et al., 2020; Chen et al., 2021).

These contextual considerations align with two relevant lines of thought: (a) the suggestion regarding the role and relevance of potential moderators that explain the intention-behavior relationship and related variables (Rhodes and Smith, 2006; Faries, 2016; Rhodes et al., 2021), and (b) recent evidence, as well as recent implications, of the potential importance of the intensity traits agreement/disagreement with current training on relevant outcomes (Teixeira et al., 2021a,b). Particularly, regarding traits agreement/disagreement, the first studies to address this hypothesis showed that in a large sample of health club exercisers, preference, tolerance, or both, in agreement with current exercise intensity, depict higher exercise frequency, subjective vitality, and habit, and generally more positive associations with well-being variables (Marques et al., 2021; Teixeira et al., 2021b). These results, among other previous suggestions, tend to reflect the moderation role of the intensity traits agreement/disagreement in affective associated variables. With this in mind, the main aim of this cross-sectional exploratory study was to test the moderation effect of the intensity traits agreement/disagreement in three relevant outcomes of exercise enjoyment: exercise habit, intention to continue exercising, and exercise frequency. It was hypothesized that the intensity traits agreement with current training intensity should positively moderate the relation between enjoyment and proposed outcomes variables, and that traits disagreement would present negative or non-significant effects (Teixeira et al., 2021a). Considering previous suggestions of the possible relevance of these traits as moderators in exercise adherence variables, the present study adds new insights on how to approach the intention-behavior gap from an intensity perspective and provides health club professionals with new lines of reasoning in their exercise prescription and counseling.

## Materials and Methods

### Participants and Procedures

In the present study 273 exercisers (male = 127; *M*_age_ = 36.21; *SD* = 11.29) enrolled in nine health clubs voluntarily completed a battery of questionnaires (general sociodemographic questions and psychometric scales; see instruments section).

Participants had a mean of 12.45 (*SD* = 11.73) years of practice in health clubs and were enrolled in individual activities (44%), group classes (31%), aquatic activities (11%), or a combination of these activities (14%). In order to be able to participate, exercisers had to be ≥ 18 years, be enrolled in Portuguese health clubs, and had to have had a minimum of 60 min of weekly practice in the last 3 months. Due to some technical issues, some data on exercise habit were lost, and the analysis for this variable was conducted with 215 participants.

The present work is part of an ongoing research project on the quality of the subjective exercise experience in health clubs. For the development of this study, approval from the Ethics Committee of the Faculty of Physical Education and Sport of Lusófona University was obtained. Later, health club managers were contacted and written approval for study development was requested. The sample of health clubs was chosen by convenience and represented Portuguese middle market and premium market segments (Pedragosa and Cardadeiro, 2020). After approval, questionnaires were made available at the club’s reception desk in two forms: through a QR-code with a link to a Google forms questionnaire, or in a physical format. Answers were obtain equitably in both formats (QR-code = 133; physical format = 140). Written consent was requested before data collection in both formats to ensure that participants understood study purposes and expected participation. For that matter, an explanation letter was provided, emphasizing that the participation would be voluntary, the data would be treated with confidentiality, and that the participant could cease to participate at any moment without any repercussions. A contact of one of the researchers was also made available to allow for additional clarifications. All the procedures were developed according to the Helsinki Declaration and its later amendments.

### Measures

#### Preference for and Tolerance of Exercise Intensity (Original: PRETIE-Q; Ekkekakis et al., 2005; Portuguese Version: PRETIE-Q-PT; Teixeira et al., 2021b)

For the intensity traits level of agreement, two questions developed to complement the 10-item (five items for each factor) instrument version of PRETIE-Q-PT were used. The questions, “The intensity of my training is in accordance with my preference” and “The intensity of my training is in accordance with my tolerance”, were answered and coded with 0 (not in agreement/disagreement) or 1 (in agreement) as in the work of Teixeira et al. (2021a).

#### The Physical Activity Enjoyment Scale (Original: PACES; Kendzierski and DeCarlo, 1991; PACES Portuguese Version: Teques et al., 2017; Rodrigues et al., 2021b)

The PACES is an 8-item scale that assesses the level of exercise enjoyment (e.g., *“It is fun”*) using a 7-point bipolar Likert scale ranging from 1 (Totally disagree) to 7 (Totally agree). The question used was *“How do you feel at the moment about the exercise you are doing?”* The scores for enjoyment were obtained through the sum of the eight items.

#### Self-Report Behavioral Automaticity Index (Original: SRBAI; Gardner et al., 2012; SRBAI Portuguese Version: Rodrigues et al., 2021a)

The SRBAI is a 4-item scale that measures behavioral habits related to exercise. The statement “*Exercise is something*” preceded the four items (e.g., *“I start doing before I realize I am doing it”*). Participants rated how true each statement was for them on a 7-point bipolar Likert scale, ranging from 1 (“Totally disagree”) to 7 (“Totally agree”). The exercise habit score was obtained through the sum of all the items.

#### Intention to Continue Exercising

Three items were used to assess intention to continue exercising after 6 months, which followed Ajzen (2006) and previous related studies recommendations and applications (e.g., Rodrigues et al., 2020). The items had been previously translated using methodological recommendations (Brislin, 1970, 1980). The items *“I will continue to exercise in the next 6 months as I currently do or in a very similar way (same type, frequency, duration, and intensity),” “I will continue to practice physical exercise in the next 6 months as I currently practice or in a very similar way (same type, frequency, duration, and intensity),”* and *“I plan to continue practicing physical exercise in the next 6 months as I do today or in a very similar way (same type, frequency, duration, and intensity),”* were answered in a 7-point bipolar Likert scale ranging from 1—“Absolutely not” to 7—“Absolutely yes.” The behavioral intention score was obtained through the sum of all the items.

#### Exercise Frequency

Exercise frequency was self-reported by answering to *“On average, how many workouts do you do per week in the club?”*

### Statistical Analysis

Descriptive analysis and bivariate correlation were calculated for all variables. In cases with more than 5% of absent data (2.2%), participants were removed prior to statistical analysis (except for habit in the 58 participants where these responses were not obtained). No imputation procedures were developed. Calculations were performed using SPSS Statistics v. 23.0 for Windows (IBM Co., United States), setting statistical significance at *p* < 0.05.

For moderation purposes the SPSS PROCESS V. 3.5. macro was used and Hayes’ (2018) recommendations were followed. To analyze the different moderation models hypothesized, the model 1 specification (i.e., single moderator testing between the independent and dependent variable) was chosen. This feature allows for conducting the analysis and interpretation if the estimation of the effect of the independent variable (enjoyment) on a dependent variable (e.g., intention) presents changes in size, sign, or strength of the effect (i.e., moderated; intensity-trait agreement/disagreement). Additionally, mean center for construction of products was used for all variables that define products. This procedure allows for the simplification of path analysis and significance interpretations without changing the moderation and interaction scores and effects. Finally, a bootstrap with 5,000 samples was used for CI95% intervals estimation, and significant effects were considered if CI did not encompass zero.

## Results

Data were initially screened for analysis assumptions and no issues were detected. Descriptive, reliability, and correlation analyses results are depicted in Table 1. All tested variables presented positive associations (all *p* < 0.01), ranging from weak to moderate strength of associations. For enjoyment, habit, and intention, results depicted scores above the constructs mid-point. Preference or tolerance agreement were present in 85.5 and 88.7% of exercisers, respectively. Reliability through Cronbach’s alpha indicated excellent scores.

**TABLE 1:** Descriptive, reliability, and correlation analysis of studied variables.

	α	Score range	M	SD	1	2	3	4
1. Enjoyment	0.94	8–56	46.28	8.16	1			
2. Habit	0.91	4–28	17.53	6.65	0.519***	1		
3. Intention	0.94	3–21	18.89	3.29	0.383***	0.176**	1	
4. Exercise frequency	–	1–7	2.99	1.26	0.563***	0.320***	0.298***	1

		**0 (Disagreement)**		**1 (Agreement)**				

Preference	–	14,50%		85,50%				
Tolerance	–	11,30%		88,70%				

α, Cronbach’s alpha; M, mean; SD, standard deviation; **p < 0.01; ***p < 0.001.

**FIGURE 1:**
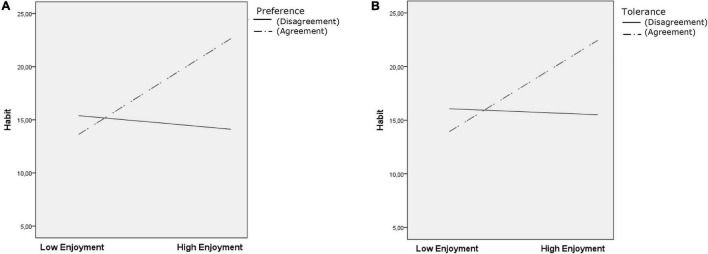
Conditional effects analysis for the preference and tolerance levels of agreement/disagreement on exercise habit.

**FIGURE 2:**
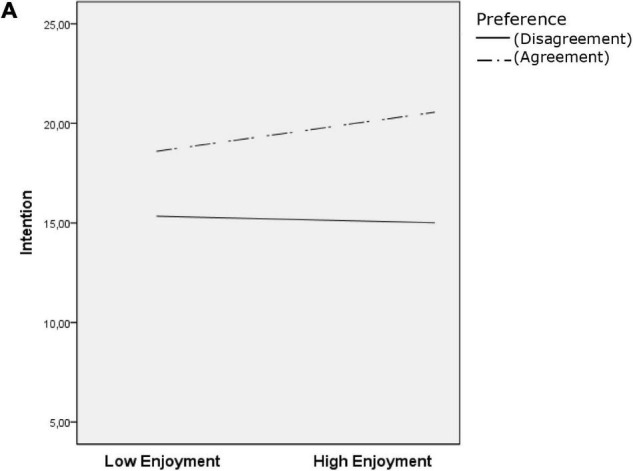
Conditional effects analysis for the preference level of agreement/disagreement on intention to continue exercising.

**FIGURE 3:**
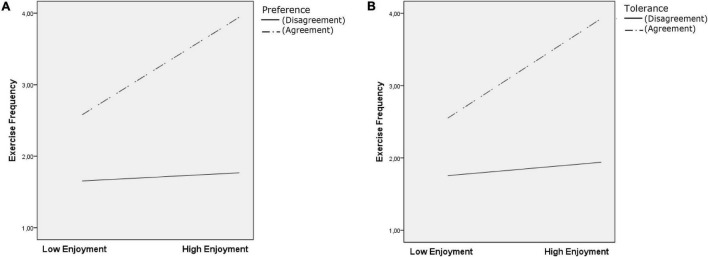
Conditional effects analysis for the preference and tolerance levels of agreement/disagreement on exercise frequency.

Regarding moderation analysis (Table 2), preference acted as a moderator in the three models tested (all independent variables × Moderator *p* < 0.05) (Figure 1a, 2a, and 3a). Tolerance moderated the habit and frequency models (Habit × Moderator *p* < 0.001 Figure 1b; Frequency × Moderator *p* = 0.005) (Figure 3b). Test for conditional effects (i.e., probing interactions) (Table 2) supported previous moderation results for preference agreement in all models (all *p* < 0.001) and tolerance agreement in the two previous models (habit and frequency; both *p* < 0.001). No significant effect emerged from preference or tolerance disagreement in all the tested models.

**TABLE 2:** Moderation and interaction analysis of preference and tolerance agreement/disagreement.

Habit Model											

	Coeff.	*t*	*p*	LLCI	ULCI		Coeff.	*t*	*p*	LLCI	ULCI
Enjoyment	0.47	0.05	< 0.001	0.364	0.578	Enjoyment	0.47	8.85	< 0.001	0.364	0.573
Preference	2.48	1.40	0.078	−0.287	5.250	Tolerance	1.61	1.13	0.262	−1.206	4.418
Interaction	0.64	0.16	< 0.001	0.334	0.954	Interaction	0.57	3.36	< 0.001	0.234	0.899
*R*^2^ = 0.32; MSE = 30.80						*R*^2^ = 0.30; MSE = 31.58					

	***R*^2^-change**	***F***	**df1**	**df2**	***p***		***R*^2^-change**	***F***	**df1**	**df2**	***p***

IV × Moderator	0.05	16.719	1	211	< 0.001	IV × Moderator	0.04	11,316	1	211	< 0.001

**Conditional effects**	**Effect**	***t***	***p***	**LLCI**	**ULCI**	**Conditional effects**	**Effect**	***t***	***p***	**LLCI**	**ULCI**

Preference = 0	−0.08	−0.546	0.586	−0.368	0.209	Tolerance = 0	−0.035	−0.222	0.825	−0.349	0.278
Preference = 1	0.56	9.670	< 0.001	0.449	0.679	Tolerance = 1	0.532	9.474	< 0.001	0.421	0.643

**Intention Model**											

	**Coeff.**	***t***	***p***	**LLCI**	**ULCI**		**Coeff.**	***t***	***p***	**LLCI**	**ULCI**

Enjoyment	0.10	4.52	< 0.001	0.057	0.145	Enjoyment	0.13	5.88	< 0.001	0.089	0.178
Preference	4.18	6.89	< 0.001	2.987	5.38	Tolerance	2.01	2.99	< 0.001	0.688	3.33
Interaction	0.14	2.45	0.015	0.028	0.256	Interaction	0.02	0.29	0.769	-0.130	0.176
*R*^2^ = 0.27; MSE = 7.75						*R*^2^ = 0.17; MSE = 8.83					

	***R*^2^-change**	***F***	**df1**	**df2**	***p***		***R*^2^-change**	***F***	**df1**	**df2**	***p***

IV × Moderator	0.02	5.99	1	269	0.015	IV × Moderator	< 0.001	0.086	1	269	0.769

**Conditional effects**	**Effect**	***t***	***p***	**LLCI**	**ULCI**	**Conditional effects**	**Effect**	***t***	***p***	**LLCI**	**ULCI**

Preference = 0	−0.02	−0.385	0.701	−0.124	0.083	Tolerance = 0	–	–	–	–	–
Preference = 1	0.12	4.962	< 0.001	0.073	0.170	Tolerance = 1	–	–	–	–	–

**Frequency Model**											

	**Coeff.**	***t***	***p***	**LLCI**	**ULCI**		**Coeff.**	***t***	***p***	**LLCI**	**ULCI**

Enjoyment	0.07	9.399	< 0.001	0.058	0.089	Enjoyment	0.08	10.14	< 0.001	0.062	0.092
Preference	1.43	6.739	< 0.001	1.012	1.845	Tolerance	1.27	5.68	< 0.001	0.832	1.716
Interaction	0.08	3.829	< 0.001	0.038	0.117	Interaction	0.07	2.83	0.005	0.023	0.125
*R*^2^ = 0.41; MSE = 0.945						*R*^2^ = 0.39; MSE = 0.98					

	***R*^2^-change**	***F***	**df1**	**df2**	***p***		***R*^2^-change**	***F***	**df1**	**df2**	***p***

IV × Moderator	0.03	14.661	1	269	< 0.001	IV × Moderator	0.02	8.020	1	269	0.005

**Conditional effects**	**Effect**	***t***	***p***	**LLCI**	**ULCI**	**Conditional effects**	**Effect**	***t***	***p***	**LLCI**	**ULCI**

Preference = 0	0.01	0.384	0.699	−0.029	0.043	Tolerance = 0	0.01	0.462	0.645	−0.037	0.060
Preference = 1	−0.08	9.873	< 0.001	0.068	0.101	Tolerance = 1	0.09	10.736	< 0.001	0.070	0.101

*p, significance value; LLCI, lower level confidence interval; ULCI, upper level confidence interval; MSE, mean square error.*

## Discussion

Given the need to explore the factors that can account for a better understanding of the intention-behavior gap in health clubs settings, and considering the emergence of hedonic assumptions related to exercise adherence, this cross-sectional study aimed to test the moderation effect of the intensity traits agreement/disagreement in three relevant outcomes of exercise enjoyment: exercise habit, intention to continue exercising, and exercise frequency. Descriptive results indicate above mid-point scores for all psychological variables and an average weekly exercise frequency of 2.99 (*SD* = 1.26). Enjoyment presented positive associations with all variables (all *p* < 0.001). Moderation tests results indicate that enjoyment positively predicted all studied outcomes (all *p* < 0.001), and that the intensity traits agreement (except tolerance agreement > intention) positively moderated all the associations. No significant moderation role emerged from the intensity traits disagreement. The present results are aligned with the previous hypothesis suggesting that the intensity traits agreement with current training intensity might positively moderate the relation between enjoyment and proposed outcomes variables, and that traits disagreement would present negative or non-significant effects.

Health clubs and other related settings have struggled to keep exercisers enrolled in their activities. Given the myriad of possible factors that can emerge that justify this problem, literature tends to suggest that interpersonal behaviors (particularly professional-exerciser relationships) have an important role that can account for some of the dropouts and lack of engagement (Rodrigues et al., 2018, 2019; Rand et al., 2020). Although dropout and lack of commitment can occur in all exercisers and at any point of their exercise experience, this tends to happen primarily in the first months of practice (Sperandei et al., 2016; International Health Racquet and Sportsclub Association, 2020; Rand et al., 2020). Thus, a better understanding of professional behaviors that can help reduce these issues can prove to have a relevant role in adherence and, ultimately, on the general population’s health.

It can be assumed that in supervised activities the professional should develop his intervention aiming to address not only individual’s needs, *but also* individual’s preferences. Like that, this may help beginner exercisers develop a more adequate relation between exercise and bodily feelings, thus aiming to improve affect processing (automatic and reflective precursors), which may have consequences on enjoyment development, habit, and intentions to continue exercising (Williams and Evans, 2014; Stevens et al., 2020; Strobach et al., 2020; Cheval and Boisgontier, 2021). This also applies to more experienced exercisers, for instance, as results of immediate affective response, but particularly, post-behavior affective response, may influence reflective affect processing with consequences in exercise enjoyment (Stevens et al., 2020). Being enjoyment a self-determined factor commonly associated with exercise adherence (Nielsen et al., 2014; Calder et al., 2020; Chen et al., 2020), exercise supervision and prescription characteristics that may be aligned with the promotion of the pleasurable component of enjoyment should depict positive associations with exercise behavior (Rodrigues et al., 2020; Stevens et al., 2020).

Moreover, enjoyment perception of a given activity is not expected to be a static process. The characteristics of what is considered enjoyable may reflect a wide array of subjective aspects, and can be manifested in distinct ways (Rodrigues et al., 2021b). As seen in this study results, enjoyment positively predicted exercise habit, intention to continue exercising, and exercise frequency, aligning with previous empirical assumptions. More interestingly, the perception of agreement of each intensity-trait presented a moderation effect, albeit slightly favoring preference. As seen, interaction results showed a better model fit in frequency models (preference: *R*^2^ = 0.41; tolerance: *R*^2^ = 0.39), higher moderation effect in habit model (preference: independent variable × moderator *R*^2^_change_ = 0.05; tolerance: independent × moderator *R*^2^_change_ = 0.04), and results not so expressive or significant (tolerance > intention) in the intention models, which highlights a distinct role of enjoyment and traits agreement on the outcome variables. Additionally, results seem to be in line with the hypothesis presented previously; when exercisers perceived an alignment with their intensity traits, there is a suggestion that they will exercise more frequently, develop higher exercise habit, and sustain a higher intention to continue practicing. Regarding intention, it must be noted that the mean scores depicted in Table 1 show a near-maximum value (*M* = 18.89; max = 21), thus suggesting a possible ceiling effect that can account for (and in case of tolerance, justify the absence of) the moderation effects. Moreover, considering the sample years of training experience (*M* = 12.45), it is somehow expected that several individuals have integrated the exercise practice in their life, thus reflecting higher means in the outcome variables which could affect moderation magnitude. Still, on the intention outcomes, the results may also coincide with some of the PAAM model assumptions. Given that intention tends to reflect explicit processes, and thus, less dependent on automatic affective processing and associations, changes promoted by the intensity traits agreement which are expected to influence the affective component of enjoyment, may express themselves with lower effects. All in all, these may justify the lower model fit and less relevant scores obtained with exercise intention.

The previous model’s results and contextual interpretations may be reinforced when considering that no interaction emerged with the intensity traits disagreement. It is possible that long-term exercisers have found throughout the time the adequate activities and intensities that make them feel pleasure and consider enjoyable, or that the exercise professionals do account for individual preferences when developing the activities. Data from Table 1 suggest that, considering that exercisers report that preference (85.50%) and tolerance (88.70%) are in agreement with the activities they engage in. This hypothesis may justify a future need for differentiating the intensity traits moderation role in distinct experience groups (e.g., novice vs. experienced) and with higher disagreement perceptions, thus shedding some light on the possible inferences in the exercisers more prone to dropout. To date, two studies have supported this premise, and showed that traits disagreement (individually or jointly) do have differentiated outcomes in several behavior outcomes (Marques et al., 2021; Teixeira et al., 2021b), but more research is needed to clarify this assumption.

Contextually, present study results suggest that assessing and tailoring exercise prescription and supervision aiming to contemplate intensity delivery and exposure may augment future exercise behavior. This may justify the need to reflect on exercise evaluation processes aiming to target preferences identification and intra- and post-session affective assessments, as for supervision techniques/methods that can account for better counseling and activities adjustments.

### Limitations and Future Studies

The present exploratory study, despite its strengths, has some limitations that should be acknowledged for an adequate interpretation and future implications. Firstly, the study design (cross-sectional), as for the characteristics of the sample, could depict a survivor bias. When considering the high exercise experience in the study participants, it is expected that the sample size of the exercisers more prone to dropout (0 to 6 months) would be lower, justifying taking caution when interpreting the results, but also redirecting further research efforts on this topic. Moreover, the current sample had a higher percentage of preference or tolerance agreement, and high enjoyment scores, which may bias the results toward a specific subgroup of exercisers. Additional efforts should be made to test these assumptions with a longitudinal approach, and in a more heterogeneous sample of exercisers, particularly considering exercise experience and distinct agreement/disagreement levels.

Secondly, it must be noticed that no moderation model was tested with both intensity traits simultaneously nor it was considered specific agreement/disagreement subgroups (e.g., preference and tolerance agreement subgroup; not preference but tolerance agreement subgroup). Individuals may present an agreement between preference and current training regimen, but also a disagreement with tolerance. These combinations should be of interest for future research focused on individual responses to exercise intensity and related subjective exercise experiences.

Thirdly, exercise frequency was obtained through self-report and with only one item. A relevant improvement in the understanding of this outcome may be achieved with additional questions (e.g., framed by time periods; duration), and particularly through objective measures (e.g., history of gym access; direct observation).

In conclusion, the results presented a moderation effect of exercise intensity traits agreement on three relevant enjoyment outcomes: exercise habit, intention to continue exercising, and exercise frequency. No relevant results emerged from intensity traits disagreement. Present study results suggest that assessing and tailoring exercise prescription and supervision aiming to contemplate intensity delivery and exposure may augment future exercise behavior.

## Data Availability Statement

The raw data supporting the conclusions of this article will be made available by the authors, without undue reservation.

## Ethics Statement

The studies involving human participants were reviewed and approved by Ethics committee of the Faculty of Physical Education and Sport, ULHT. The patients/participants provided their written informed consent to participate in this study.

## Author Contributions

DT, FR, LC, and DM contributed to conception design of the study. DT performed the statistical analysis and wrote the first draft of the manuscript. All authors contributed to manuscript revision, read, and approved the submitted version.

## Conflict of Interest

The authors declare that the research was conducted in the absence of any commercial or financial relationships that could be construed as a potential conflict of interest.

## Publisher’s Note

All claims expressed in this article are solely those of the authors and do not necessarily represent those of their affiliated organizations, or those of the publisher, the editors and the reviewers. Any product that may be evaluated in this article, or claim that may be made by its manufacturer, is not guaranteed or endorsed by the publisher.
