# Corrigendum: Enjoyment as a predictor of exercise habit, intention to continue exercising, and exercise frequency: the intensity traits discrepancy moderation role

**DOI:** 10.3389/fpsyg.2024.1417755

**Published:** 2024-05-06

**Authors:** Diogo S. Teixeira, Filipe Rodrigues, Luis Cid, Diogo Monteiro

**Affiliations:** ^1^Faculty of Physical Education and Sport (ULHT), Lusófona University of Humanities and Technologies, Lisbon, Portugal; ^2^Research Center in Sport, Physical Education, and Exercise and Health (CIDEFES), Lisbon, Portugal; ^3^ESECS, Polytechnic of Leiria, Leiria, Portugal; ^4^Quality of Life Research Center (CIEQV), Santarém, Portugal; ^5^Research Center in Sport, Health and Human Development (CIDESD), Vila Real, Portugal; ^6^Sport Science School of Rio Maior (ESDRM), Polytechnic Institute of Santarém, Santarém, Portugal

**Keywords:** exercise, intensity, enjoyment, intention, habit, moderation

In the published article, there was an error in the Funding statement. The indication that this research was supported by national funds through the Portuguese Foundation for Science and Technology, I.P., under the project UID/DTP/04748/2020, was incorrect. The correct Funding statement appears below.

